# A Clinically Applicable Nomogram for Live Birth Prediction After IVF: The Zubeyde Hanim Model

**DOI:** 10.3390/jcm15031077

**Published:** 2026-01-29

**Authors:** Pınar Karaçin, Runa Özelçi, Enes Kumcu, Dilek Kaya Kaplanoğlu, Serdar Dilbaz, Yaprak Üstün

**Affiliations:** 1 Department of Gynecology, Dr Abdurrahman Yurtaslan Ankara Oncology Training and Research Hospital, Ankara 06200, Türkiye; 2 Department of Gynecology, Etlik City Hospital, Ankara 06200, Türkiye; runakara@gmail.com (R.Ö.); enes_kumcu@hotmail.com (E.K.); sdilbaz@gmail.com (S.D.); 3 Department of Gynecology, Adana Yüreğir State Hospital, Adana 01200, Türkiye; dilekkaplanoglu@gmail.com; 4 Department of Gynecology, İzmir City Hospital, İzmir 35200, Türkiye; ustunyaprak@yahoo.com

**Keywords:** embryo quality, in vitro fertilization, live birth, nomogram, prediction model

## Abstract

**Objective:** In this study, we aimed to develop and internally validate a clinically applicable nomogram for predicting live birth following in vitro fertilization (IVF) using routinely available clinical and embryological parameters. **Methods:** This retrospective study was conducted at a single tertiary IVF center. Women undergoing IVF/ICSI were included if their baseline demographic and clinical data were available, they had undergone at least one fresh or frozen–thawed embryo transfer, and they had a known live birth outcome. Women with cycles without embryo transfer and those missing key outcome data were excluded from the analysis. As a result, a total of 2119 IVF/ICSI treatment cycles resulting in embryo transfer were included in the analysis. To identify independent predictors of live birth, multivariable logistic regression analysis was performed. **Results:** Among the 2119 treatment cycles analyzed, 541 resulted in live birth (25.5%). Multivariable logistic regression with backward stepwise selection identified female age (OR: 0.959, *p* < 0.001), high embryo quality (OR: 2.752, *p* < 0.001), day of embryo transfer (day 5 vs. day 3, OR: 1.427, *p* = 0.001), and endometrial thickness on the day of transfer as independent predictors of live birth (OR: 1.086, *p* < 0.001). These variables were incorporated into a nomogram (the Zübeyde Hanim IVF Nomogram) to estimate individualized live birth probability. The model demonstrated acceptable discrimination, with a bootstrap-corrected area under the receiver operating characteristic curve (AUC) of 0.64 (95%CI: 0.61–0.66), and it showed satisfactory calibration across deciles of predicted risk. **Conclusions:** The Zubeyde Hanim IVF Nomogram provides an individualized and clinically practical tool for predicting live birth following IVF treatment. Based on routinely available parameters, this model may assist clinicians in patient counseling and treatment planning.

## 1. Introduction

Assisted reproductive technologies (ARTs) have evolved substantially in recent decades, resulting in improved pregnancy outcomes and expanding access to infertility treatment worldwide [1]. Nevertheless, despite the technological progress that has been made in ovarian stimulation, laboratory conditions, and embryo culture, the likelihood of achieving a live birth following in vitro fertilization–embryo transfer (IVF-ET) remains highly heterogeneous among patients and treatment cycles [1]. From both a clinical and patient-centered perspective, live birth represents the most relevant and definitive outcome of IVF, surpassing intermediate endpoints such as biochemical or clinical pregnancy [2]. Consequently, identifying reliable predictors of live birth and integrating them into clinically applicable prediction models has become a priority in contemporary reproductive medicine.

IVF success is determined by a complex interaction of maternal, paternal, embryological, and treatment-related factors [3]. Female age remains the strongest and most consistently reported predictor of live birth, reflecting age-dependent declines in oocyte competence and increasing rates of chromosomal abnormalities [3,4]. However, chronological age alone provides an incomplete assessment of reproductive potential. Ovarian reserve markers, particularly anti-Müllerian hormone (AMH) and antral follicle count (AFC), are widely used to estimate quantitative ovarian response, yet their independent ability to predict live birth is limited and context-dependent [5].

Beyond ovarian reserve, embryological parameters—including embryo morphology, blastocyst development, and stage of embryo transfer—have demonstrated associations with implantation and live birth outcomes [6,7]. However, these parameters are influenced by inter-observer variability, laboratory-specific grading systems, and center-dependent practices, which restrict their universal applicability as standalone predictors [8]. Similarly, treatment-related factors such as stimulation protocols, gonadotropin dose, and endometrial preparation strategies contribute to outcome variability but rarely offer consistent predictive power when evaluated in isolation [9].

In routine clinical practice, counseling regarding IVF success often relies on single predictors or clinician experience rather than integrated, quantitative risk assessment. This approach may lead to inaccurate prognostication, unrealistic patient expectations, and suboptimal individualization of treatment strategies. With the growing availability of large IVF databases, there is increasing interest in multivariable prediction models that better reflect the multifactorial nature of live birth outcomes [10,11].

Several prognostic models and scoring systems for IVF outcomes have been proposed over the past decade. While some have demonstrated moderate discrimination, many are limited by their small sample sizes, restricted patient populations, or reliance on early-cycle outcomes such as oocyte yield or clinical pregnancy rather than live birth [12,13]. Importantly, predictors of ovarian response or implantation do not necessarily translate into predictors of live birth, as pregnancy loss and obstetric factors introduce additional biological complexity [14]. As a result, outcome-specific modeling focused explicitly on live birth has gained increasing attention.

Another methodological challenge in IVF research is the presence of repeated treatment cycles within the same patient. Many individuals undergo multiple IVF attempts, leading to non-independence of observations that can bias traditional regression analyses if not adequately addressed [10]. Predictive models that incorporate real-world treatment trajectories and repeated cycles are therefore more likely to generate clinically meaningful and generalizable estimates.

Developing prediction models based on large, single-center cohorts offers distinct advantages. Standardized clinical protocols, consistent laboratory conditions, and uniform outcome definitions reduce heterogeneity and strengthen internal validity [15]. At the same time, the inclusion of consecutive treatment cycles over an extended period allows for the evaluation of fresh and frozen embryo transfers, diverse stimulation strategies, and cumulative clinical experience, thereby improving real-world applicability.

This study aimed to develop and internally validate a multivariable predictive model for live birth following IVF-ET using data from approximately 2000 treatment cycles performed at a tertiary reproductive medicine center. By integrating readily available demographic, clinical, and laboratory parameters, we sought to construct a clinically applicable prediction tool reflective of routine practice. Such a model may enhance individualized patient counseling, support evidence-based decision-making, and contribute to personalized treatment planning in modern IVF care.

## 2. Methods

### 2.1. Study Design and Population

This single-center retrospective cohort study was conducted at a tertiary IVF center. All consecutive IVF/ICSI cycles performed between 2007 and 2023 were screened for eligibility. Cycles resulting in embryo transfer and with complete follow-up data for live birth outcome were included.

A total of 2119 treatment cycles were eligible for analysis. The primary outcome was live birth, defined as the delivery of at least one live-born infant beyond 24 weeks of gestation. Each cycle was treated as an independent observation. In patients undergoing more than one treatment cycle, each cycle was analyzed separately, consistent with previously published IVF prediction models.

Model development and reporting followed the TRIPOD guidelines.

### 2.2. Inclusion and Exclusion Criteria

#### 2.2.1. Inclusion Criteria

- Women undergoing IVF/ICSI were included in this study if they had the following:
  ○ Available baseline demographic and clinical data;
  ○ At least one embryo transfer (fresh or frozen–thawed);
  ○ Known live birth outcome.

#### 2.2.2. Exclusion Criteria

The exclusion criteria comprised the following:

- Cycles without embryo transfer;
- Cycles with missing key outcome data (live birth status);
- Cycles involving experimental stimulation or laboratory protocols;
- Cycles using donor oocytes or donor sperm.

### 2.3. Clinical and Laboratory Data Collection

The variables that were extracted from the institutional electronic medical records and IVF laboratory database are detailed in the following subsections.

#### 2.3.1. Demographic and Baseline Characteristics

Female age (years), body mass index (BMI, kg/m^2^), and duration of infertility (months) were recorded as baseline demographic variables. Infertility type was classified as primary infertility, defined as the absence of any prior clinical pregnancy, or secondary infertility, defined as a history of at least one previous clinical pregnancy, regardless of outcome.

#### 2.3.2. Infertility Diagnosis

Infertility etiology was classified into male factor infertility, tubal factor infertility, ovulatory or hormonal disorders, polycystic ovary syndrome (PCOS), and unexplained infertility.

#### 2.3.3. Baseline Hormonal Assessment

Baseline hormonal assessment included basal day 3 serum follicle-stimulating hormone (FSH) and estradiol (E2) levels. All hormone measurements were performed using standardized immunoassay techniques in the same institutional laboratory.

### 2.4. Controlled Ovarian Stimulation and Laboratory Procedures

Controlled ovarian stimulation was conducted using either a GnRH agonist long protocol or a GnRH antagonist protocol, selected according to clinician preference and individual patient characteristics. Oocyte retrieval was performed 34–36 h after ovulation was triggered. Fertilization was achieved predominantly through intracytoplasmic sperm injection (ICSI), in accordance with the routine laboratory practice of the center.

### 2.5. Embryo Culture and Transfer

Embryos were cultured under standard laboratory conditions and transferred either on day 3 at the cleavage stage or on day 5 at the blastocyst stage. Both fresh and frozen–thawed embryo transfer cycles were included in the analysis. Endometrial thickness was measured using transvaginal ultrasonography on the day of embryo transfer.

#### Embryo Quality Assessment

Embryo morphology was assessed according to routine laboratory grading systems, in line with the principles of the 2011 Istanbul consensus on embryo assessment [8]. For analytical purposes, embryos were categorized as high-quality (Grade 1 or Grade 2) or low-quality (Grade 3 or Grade 4). The variable “high-quality embryo transfer” was defined as a binary variable (yes/no) indicating whether at least one high-quality embryo was transferred in the cycle based on standardized embryo grading criteria. In cycles where multiple embryos were transferred, embryo quality classification was based on the presence of at least one high-quality embryo.

### 2.6. Outcome Measure

The primary outcome was live birth, defined as the birth of a living neonate after ≥24 weeks of gestation.

During the preparation of this manuscript, support was received from artificial intelligence-based tools, including Jenni AI (Altum Inc., Wilmington, DE, USA) and ChatGPT-5 (OpenAI, San Francisco, CA, USA), solely to improve the quality and clarity of the English language and overall manuscript structure. No data analysis, statistical modeling, or interpretation of the results was performed using artificial intelligence. All scientific content and data are original. The authors critically reviewed, verified, and edited all AI-assisted outputs and take full responsibility for the accuracy, integrity, and content of this publication.

### 2.7. Statistical Analysis

Continuous variables were expressed as mean ± standard deviation or median (interquartile range), as appropriate. Categorical variables were presented as counts and percentages. To identify independent predictors of live birth, multivariable logistic regression analysis was performed. Variables considered clinically relevant and supported by the prior literature were entered into the initial model. A backward stepwise elimination approach based on the likelihood ratio test was applied to derive the final prediction model. Variables were retained in the model if they remained statistically significant or demonstrated stable clinical relevance. Candidate variables were selected a priori based on clinical relevance and the previous literature. Multicollinearity among candidate predictors was assessed prior to model fitting, and no significant collinearity was detected. A backward stepwise logistic regression approach was subsequently used to derive a parsimonious prediction model. Variables reflecting clinical treatment decisions, such as the number of embryos transferred, were not included as candidate predictors to avoid confounding by indication and to preserve model interpretability. Model discrimination was assessed using the area under the receiver operating characteristic (ROC) curve (AUC).

Internal validation was performed using bootstrap resampling with 500 iterations to estimate optimism-corrected model performance.

Model calibration was evaluated using the following:

- Calibration plots based on deciles of predicted risk;
- Comparison of observed versus predicted live birth probabilities.

Clinical utility was assessed using decision curve analysis, estimating net benefit across a range of threshold probabilities.

To address potential within-patient correlation due to repeated cycles, sensitivity analyses restricted to the first embryo transfer cycle per woman were performed. In addition, clustered marginal models using generalized estimating equations with the patient identifier as the clustering variable were fitted. To assess the robustness of the model assumptions, several sensitivity analyses were performed. These included restriction of the analysis to the first embryo transfer cycle per woman, fitting clustered marginal models using generalized estimating equations to account for within-patient correlation, and stratification by embryo transfer type (fresh versus frozen).

Based on the final multivariable model, a graphical nomogram was constructed to provide individualized predictions of live birth probability.

All statistical analyses were performed using Statistical Package for the Social Sciences (SPSS), version 25.0 (IBM Corp., Armonk, NY, USA), and supplementary analyses, including bootstrap resampling, ROC curve analyses, and figure generation, were performed using R statistical software (version 4.0.2; R Foundation for Statistical Computing, Vienna, Austria). A two-sided *p*-value < 0.05 was considered statistically significant.

## 3. Results

A total of 2119 embryo transfer cycles were included in the analysis, of which 541 (25.5%) resulted in live birth. The baseline demographic and clinical characteristics according to live birth outcome are summarized in Table 1. Women who achieved live birth were significantly younger than those who did not (29 ± 4.8 vs. 31 ± 5.6 years, *p* < 0.001) and had a shorter duration of infertility (55 ± 43.2 vs. 61 ± 50.4 months, *p* = 0.012). Antral follicle count was also higher in the live birth group (14 ± 8.1 vs. 13 ± 8.1, *p* = 0.012). No significant differences were observed between groups with respect to body mass index, male age, infertility type (primary vs. secondary), gravidity, previous live birth history, basal gonadotropin levels (FSH and LH), or basal estradiol concentrations. The distribution of infertility diagnoses was largely comparable between groups, although male factor infertility tended to be more frequent among cycles resulting in live birth (43.3% vs. 38.5%, *p* = 0.050).

Stimulation and laboratory characteristics are presented in Table 2. The use of GnRH agonist and antagonist protocols was similar between cycles with and without live birth (*p* = 0.372). Total gonadotropin dose and stimulation duration did not differ significantly between groups, with a mean stimulation length of approximately 10 days in both cohorts. Trigger-day estradiol and progesterone levels were comparable, and mean progesterone concentrations remained below clinically relevant thresholds for premature luteinization. While the total number of retrieved and mature oocytes did not differ significantly, the number of normally fertilized oocytes (2PN) was significantly higher in cycles resulting in live birth (5.4 ± 3.4 vs. 4.8 ± 3.6, *p* = 0.001).

Table 3 summarizes the baseline differences between fresh and frozen embryo transfer cycles and provides the rationale for the performance of additional sensitivity analyses. Endometrial thickness on the day of transfer was similar between fresh and frozen cycles. A significant difference in the day of embryo transfer was observed, with cleavage-stage (day 3) transfers being more frequent in fresh cycles, whereas blastocyst transfers predominated in frozen cycles (*p* < 0.001). The number of embryos transferred also differed significantly between groups, with single-embryo transfer being more common in fresh cycles and multiple-embryo transfer occurring more frequently in frozen cycles (*p* < 0.001). High-quality embryos were transferred at a higher rate in frozen embryo transfer cycles compared with fresh transfers (92.1% vs. 86.4%, *p* = 0.001).

Baseline demographic, clinical, and treatment-related characteristics of the study population were entered into the multivariable analysis as candidate predictors of live birth.

### 3.1. Multivariable Logistic Regression Analysis Results

Using a backward stepwise logistic regression approach, independent predictors of live birth were identified. The final model retained four variables as statistically significant predictors: female age, high-quality embryo transfer, day of embryo transfer (day 5 vs. day 3), and endometrial thickness on the day of transfer.

The results of the multivariable logistic regression analysis evaluating independent predictors of live birth are presented in Table 4. Female age was independently and inversely associated with live birth, with each additional year of age associated with a reduced likelihood of live birth (odds ratio [ORs] 0.959; 95% confidence interval [CI] 0.940–0.978; *p* < 0.001). The transfer of a high-quality embryo was a strong positive predictor of live birth compared with low-quality embryos (OR 2.752, 95% CI 1.857–4.077; *p* < 0.001). Blastocyst-stage transfer (day 5) was also independently associated with higher odds of live birth compared with cleavage-stage transfer (day 3) (OR 1.427, 95% CI 1.161–1.754; *p* = 0.001). In addition, greater endometrial thickness on the day of embryo transfer was associated with a modest but statistically significant increase in live birth probability (OR 1.086 per mm, 95% CI 1.042–1.132; *p* <0.001).

Other candidate variables—including body mass index, infertility type (primary vs. secondary), basal follicle-stimulating hormone levels, infertility diagnosis (including ovulatory disorders, unexplained infertility, and polycystic ovary syndrome), male factor infertility, number of normally fertilized oocytes (2PN), transfer type (fresh vs. frozen), and prior live birth history—were not retained in the final multivariable model during the backward elimination process due to the lack of independent predictive value.

### 3.2. Nomogram Development

**Figure 1:**
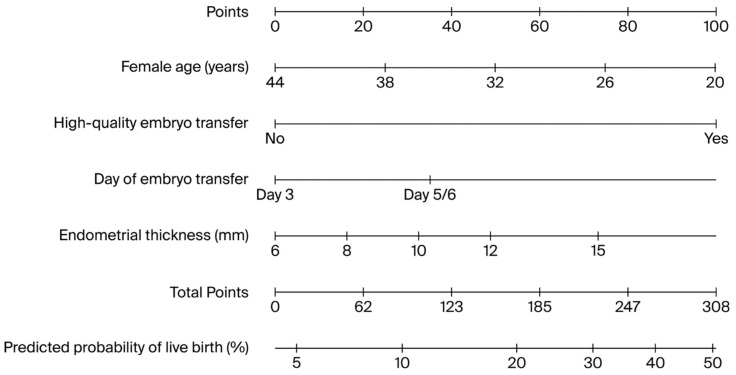
Nomogram for predicting probability of live birth.

Based on the final multivariable logistic regression model, a clinically applicable nomogram was constructed to estimate the individualized probability of live birth (Figure 1). Each predictor was assigned a weighted point value proportional to its regression coefficient, with total points corresponding to the predicted live birth probability.

### 3.3. Model Discrimination

**Figure 2:**
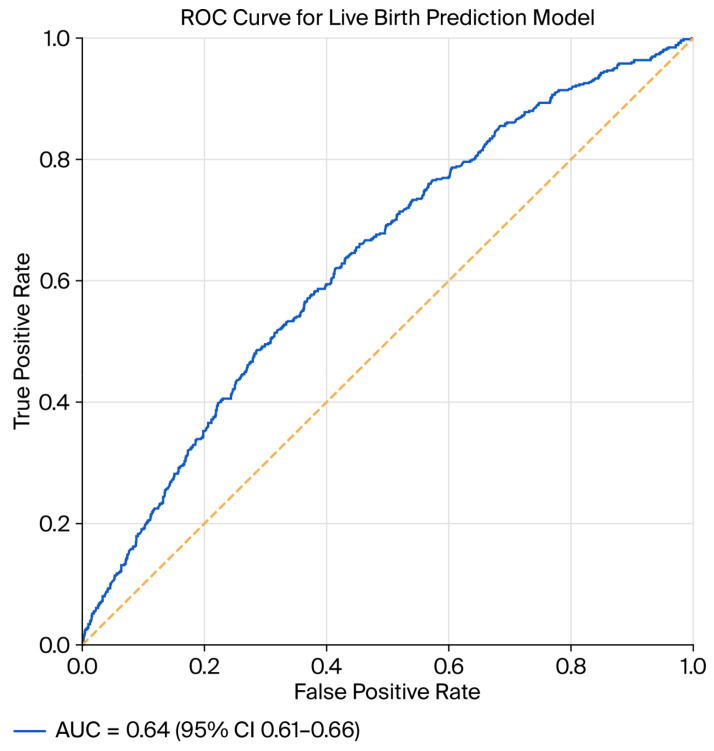
ROC curve of the final model after validation using 500 bootstrap samples. The dashed line represents the line of no discrimination (AUC = 0.5).

The discriminative performance of the model was evaluated using the area under the receiver operating characteristic curve (AUC). The model demonstrated acceptable discrimination, with an apparent AUC of approximately 0.64 (95%CI: 0.61–0.66). After internal validation using 500 bootstrap resamples, the optimism-corrected AUC remained stable, indicating minimal overfitting (Figure 2).

### 3.4. Model Calibration

**Figure 3:**
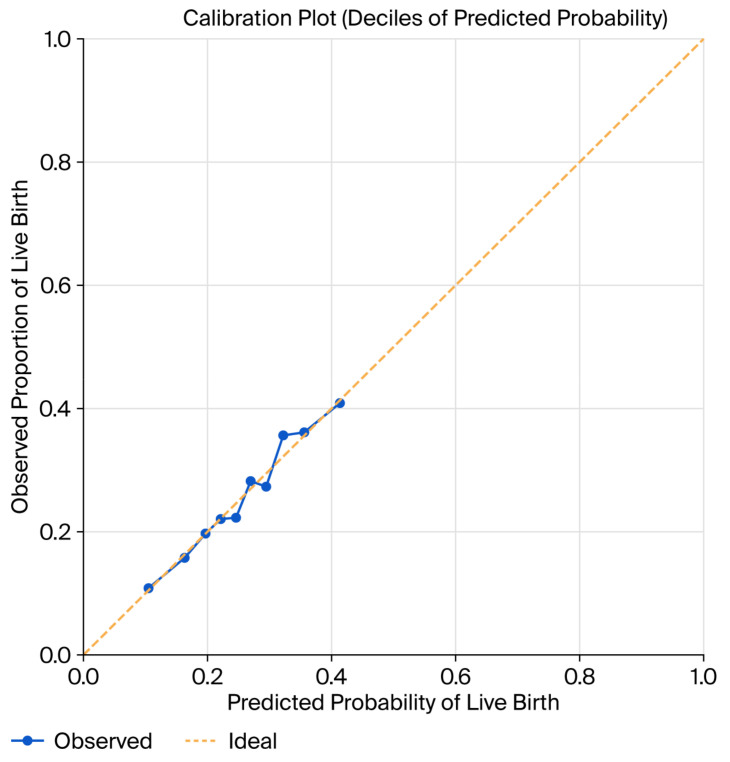
Calibration plot based on deciles of predicted probability. The dashed line indicates the ideal calibration line, representing perfect agreement between predicted and observed probabilities.

Calibration of the model was assessed using calibration plots based on deciles of predicted risk. The calibration curve demonstrated good agreement between predicted and observed live birth probabilities across the entire range of risk, with only minor deviation at extreme probability values (Figure 3). This finding suggests that the model provides reliable absolute risk estimates.

### 3.5. Decision Curve Analysis

**Figure 4:**
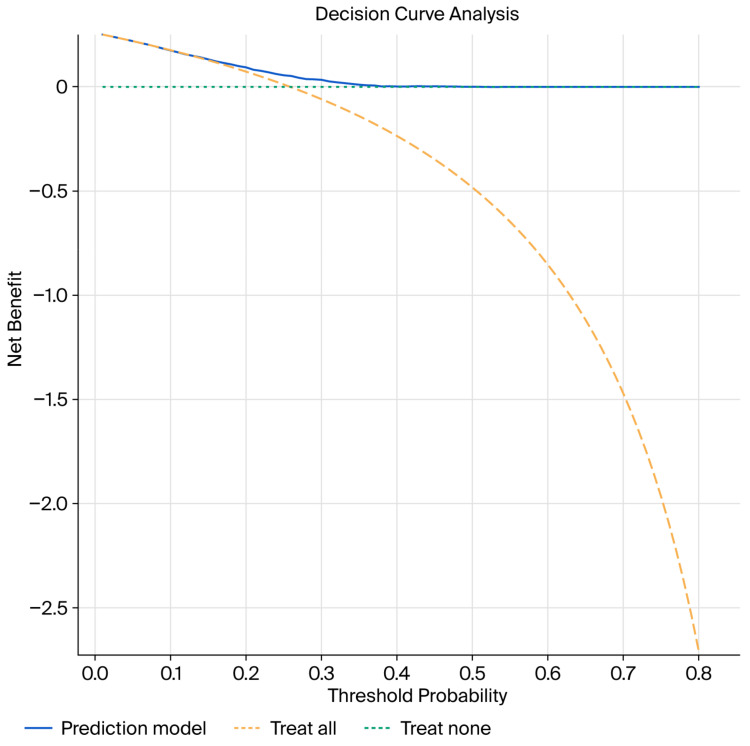
Decision curve analysis demonstrating net clinical benefit. The dashed lines represent the “treat-all” and “treat-none” strategies.

Decision curve analysis was performed to evaluate the clinical utility of the nomogram. The model demonstrated a net clinical benefit across a wide range of threshold probabilities compared with both “treat-all” and “treat-none” strategies (Figure 4), supporting its potential usefulness in individualized clinical decision-making.

### 3.6. Sensitivity Analyses

Supplementary Tables S1–S3 present the results of the sensitivity analyses, including those restricted to first cycles, clustered models, and stratification by transfer type. Specifically, Table S1 shows the results for the sensitivity analysis restricted to the first IVF cycle per woman. This analysis was performed to address potential within-patient correlation due to repeated cycles. The AUC was 0.63, 95% CI: 0.61–0.67; thus, even when limited to first cycles, the results did not change (Table S1). Model discrimination was comparable between fresh and frozen embryo transfer cycles. Stratified analyses by transfer type are presented in Supplementary Table S3.

### 3.7. Internal Validation

Internal validation using bootstrap resampling (500 iterations) confirmed the robustness of the model. Regression coefficients, AUC values, and calibration parameters showed minimal optimism, indicating that the model maintains predictive accuracy when applied to new samples drawn from the same population. Details of the bootstrap internal validation, including apparent and optimism-corrected AUC estimates, are provided in Supplementary Table S4. The apparent AUC was 0.64, 95% CI (bootstrap percentile: 0.61–0.66), and the optimism-corrected AUC was 0.63.

## 4. Discussion

In this large, single-center cohort study including 2119 IVF treatment cycles, we developed and internally validated a nomogram to predict live birth based on routinely available clinical and embryological parameters. Using a backward stepwise logistic regression approach with bootstrap validation, four variables emerged as independent predictors of live birth: female age, embryo quality, day of embryo transfer, and endometrial thickness on the day of transfer. The final model demonstrated acceptable discrimination, with a bootstrap-corrected AUC of approximately 0.64, which is comparable to previously published IVF prediction models.

Female age remained a strong and independent negative predictor of live birth in our model. This finding is entirely consistent with the established literature and reflects the well-documented age-related decline in oocyte competence, embryo developmental potential, and endometrial receptivity [16,17]. Numerous IVF prediction models and nomogram studies have consistently identified female age as one of the most influential determinants of live birth, often with effect sizes comparable to or exceeding those observed in our cohort [10,18]. Importantly, the persistence of female age as a significant predictor even after adjustment for embryo-related variables underscores its overarching biological impact, which cannot be fully mitigated by laboratory advances alone.

Embryo quality emerged as the strongest positive predictor of live birth, with cycles involving the transfer of at least one high-quality embryo demonstrating an approximate threefold increase in live birth probability. This result aligns closely with previous studies showing that morphological embryo grading remains a robust proxy for implantation and live birth potential, despite increasing interest in adjunctive technologies such as time-lapse imaging and preimplantation genetic testing [8,19]. Several prior nomogram-based prediction models have similarly identified embryo quality as a central determinant of treatment success [12,20]. Our findings reinforce the continued relevance of conventional morphology-based embryo assessment in real-world clinical practice, particularly in settings where advanced selection techniques are not universally applied.

Day 5 embryo transfer was independently associated with higher odds of live birth compared with day 3 transfer. This observation is consistent with both randomized trials and large observational studies suggesting superior implantation and live birth rates with blastocyst-stage transfer, largely due to improved embryo self-selection and enhanced synchrony with endometrial receptivity [21,22]. Several previously published IVF prediction models have incorporated transfer day as a key predictive variable and achieved results similar to ours [13,18]. Nevertheless, it is important to acknowledge that blastocyst transfer is often reserved for cycles with favorable embryological development, which may partly explain its strong association with live birth.

Endometrial thickness on the day of embryo transfer demonstrated a modest but statistically significant association with live birth. Although the clinical relevance of endometrial thickness has been debated, multiple meta-analyses and large cohort studies support the existence of a positive relationship between increasing endometrial thickness and pregnancy outcomes, particularly at lower thickness thresholds [23,24]. Our findings are in line with previous nomogram studies that retained endometrial thickness as an independent predictor, suggesting that it provides additive prognostic information beyond embryo-related factors [12,20]. Endometrial thickness remained an independent predictor of live birth and contributed meaningfully to overall model performance; however, its clinical interpretation should be considered within the context of other key predictors rather than as a standalone determinant.

Compared with earlier IVF prediction models, our nomogram offers several strengths. It focuses on live birth rather than clinical pregnancy, incorporates both fresh and frozen embryo transfer cycles, and relies exclusively on widely available clinical parameters, enhancing its applicability in routine practice. The observed AUC of 0.64 is comparable with those reported in many previously published IVF nomograms, which typically range between 0.65 and 0.75 [10,18].

The major strengths of this study include the large sample size, cycle-based analysis, focus on live birth as the primary outcome, and rigorous internal validation using bootstrap resampling. Additionally, the use of routinely collected variables enhances the generalizability and ease of implementation of the proposed nomogram. However, this study was conducted in a single tertiary referral center with highly standardized stimulation, laboratory, and embryo transfer protocols. Practices such as the high utilization of intracytoplasmic sperm injection (ICSI) and the use of a specific embryo grading system may differ across centers and could limit the direct generalizability of the model. Therefore, the performance of the proposed nomogram may vary in settings with different laboratory techniques or clinical practices. Given the single-center retrospective design, external validation in independent, preferably multi-center cohorts is mandatory before routine clinical application of this prediction model.

This study has several limitations that should be acknowledged. First, the retrospective and single-center design may limit external generalizability, and external validation in independent cohorts is warranted. Second, each cycle was treated as an independent observation, which may introduce within-patient correlation in women undergoing multiple cycles. Third, detailed embryo genetic data and advanced embryo selection tools were not available and therefore not included in the model. Anti-Müllerian hormone (AMH), an important marker of ovarian reserve, could not be included in the prediction model because AMH measurements were available for only a limited proportion of women in the cohort, which would have resulted in a substantial loss of sample size and potential selection bias. Detailed male factor parameters were not included to maintain model simplicity and applicability across different clinical settings. Dichotomizing embryo quality improved model simplicity and clinical interpretability but may have resulted in the loss of more granular prognostic information, such as the number or relative quality of transferred embryos. Given the long inclusion period, changes in laboratory techniques, stimulation strategies, and embryo culture practices over time cannot be excluded and may have influenced the outcomes. Finally, although internal validation was robust, prospective validation is required before widespread clinical adoption of this model. Although multiple cycles from some women were included, sensitivity analyses accounting for within-patient correlation yielded consistent results, supporting the robustness of the model.

## 5. Conclusions

In conclusion, we developed and internally validated a clinically practical nomogram for predicting live birth following IVF treatment. Female age, embryo quality, transfer day, and endometrial thickness were identified as key independent predictors. This model may serve as a useful tool for individualized patient counseling and treatment planning, pending external validation.

## Figures and Tables

**Table 1:** Baseline demographic and clinical characteristics (per embryo transfer).

		Total n: 2119	No Live Birth n: 1578	Live Birth n: 541	*p*-Value
Female age, years, mean ± SD		30 ± 5.5	31 ± 5.6	29 ± 4.8	<0.001
BMI, kg/m^2^ mean ± SD		26.2 ± 7.62	26.2 ± 5.01	26.4 ± 12.41	0.591
Male age, years, mean ± SD		33 ± 5.8	33 ± 6.0	33 ± 5.1	0.469
Duration of infertility, months, mean ± SD		60 ± 48.7	61 ± 50.4	55 ± 43.2	0.012
Infertility type, n (%)					
	Secondary	488 (23.0)	375 (25.8)	113 (20.9)	0.170
	Primary	1631 (77.0)	1203 (76.2)	428 (79.1)	
Gravidity, mean ± SD		0.31 ± 0.93	0.30 ± 0.99	0.32 ± 0.75	0.667
Previous live birth, mean ± SD		0.10 ± 0.40	0.10 ± 0.40	0.10 ± 0.41	0.891
Infertility diagnosis, n (%) *					
	Male factor, yes	841 (39.7)	607 (38.5)	234 (43.3)	0.050
	Tubal factor, yes	153 (7.2)	124 (7.9)	29 (5.4)	0.053
	Ovulatory disorder, yes	377 (17.8)	273 (17.3)	104 (19.2)	0.313
	PCOS, yes	262 (12.4)	187 (11.9)	75 (13.9)	0.220
	Endometriosis, yes	136 (6.4)	103 (6.5)	33 (6.1)	0.726
	Unexplained, yes	615 (29.0)	446 (28.3)	169 (31.2)	0.188
AFC, mean ± SD		13 ± 8.1	13 ± 8.1	14 ± 8.1	0.012
Basal FSH (IU/L) (cycle day 2–3), mean ± SD		8.4 ± 3.0	8.4 ± 2.4	8.3 ± 3.1	0.439
Basal LH (IU/L), (cycle day 2–3), mean ± SD		5.7 ± 3.4	5.7 ± 3.4	5.5 ± 3.3	0.382
Basal E2 (pg/mL), (cycle day 2–3), mean ± SD		48.9 ± 39.9	49.8 ± 43.1	46.3 ± 28.4	0.077

BMI: body mass index; PCOS: polycystic ovary syndrome; AFC: antral follicle count; FSH: follicle-stimulating hormone; LH: luteinizing hormone; E2: estradiol; SD: standard deviation. * Patients could have more than one diagnosis; therefore, diagnostic categories in the demographic table are overlapping and may sum to more than the total study population. The primary diagnosis was used for outcome analyses.

**Table 2:** Stimulation and laboratory characteristics.

		Total n: 2119	No Live Birth n: 1578	Live Birth n: 541	*p*-Value
Stimulation protocol, n (%)					
	GnRH agonist	1120 (52.9)	843 (53.4)	277 (51.2)	0.372
	GnRH antagonist	999 (47.1)	735 (46.6)	264 (48.8)	
Total gonadotropin dose (IU), mean ± SD		2387 ± 983.5	2378 ± 1013.6	2457 ± 869.8	0.666
Stimulation duration (days), mean ± SD		10 ± 1.7	10 ± 1.6	10 ± 1.7	0.994
Trigger-day hormones					
	E2 (pg/mL), mean ± SD	2449 ± 2101.3	2467 ± 1659.4	2396 ± 1443.1	0.438
	P (ng/mL), mean ± SD	0.82 ± 0.61	0.82 ± 0.55	0.83 ± 0.69	0.733
Oocytes retrieved, mean ± SD		11.9 ± 7.2	11.8 ± 7.4	12.1 ± 6.9	0.344
Mature oocytes, mean ± SD		9.0 ± 5.6	8.9 ± 5.8	9.3 ± 5.2	0.124
2PN (normal fertilization), mean ± SD		5.0 ± 3.6	4.8 ± 3.6	5.4 ± 3.4	0.001

GnRH: gonadotropin-releasing hormone; E2: estradiol; P: progesterone; SD: standard deviation.

**Table 3:** Embryo transfer characteristics (fresh vs. FET).

		Fresh n: 1665	FET n: 454	*p*-Value
Endometrial thickness on transfer day (mm), mean ± SD		10.5 ± 2.15	10.4 ± 2.17	0.591
Day of transfer, n (%)				
	Day 3	990 (59.5)	220 (48.5)	<0.001
	Day 5 *	675 (40.5)	234 (51.5)	
Number of embryos transferred, n (%)				
	1	1329 (79.8)	172 (37.9)	<0.001
	2	282 (16.9)	90 (19.8)	
	3	54 (3.2)	192 (42.3)	
High-quality embryo transferred, n (%)				
	No	226 (13.6)	36 (7.9)	0.001
	Yes	1439 (86.4)	418 (92.1)	

FET: frozen embryo transfer; SD: standard deviation. * Blastocyst transfers included embryos transferred on day 5 or day 6.

**Table 4:** Multivariable logistic regression analysis.

	Odds Ratio (95% Confidence Interval)	*p*-Value
Female age	0.959 (0.940–0.978)	<0.001
Quality of embryo, high vs. low	2.752 (1.857–4.077)	<0.001
Embryo transfer day, day 5 vs. day 3	1.427 (1.161–1.754)	0.001
Endometrial thickness on transfer day	1.086 (1.042–1.132)	<0.001

## Data Availability

All the data underlying this article are available in text, tables, and References (CrossRef). If further breakdown is required, such data will be shared on reasonable request to the corresponding author.

## Supplementary Materials

The following supporting information can be downloaded at https://www.mdpi.com/article/10.3390/jcm15031077/s1, Table S1: Sensitivity Analysis Restricted to First IVF Cycle per Woman; Table S2: Clustered Marginal Model Using Generalized Estimating Equations (GEE); Table S3: Model Discrimination by Embryo Transfer Type; Table S4: Bootstrap internal validation results for model discrimination.
