# Supplementary material for: A Clinically Applicable Nomogram for Live Birth Prediction After IVF: The Zubeyde Hanim Model
Source: J Clin Med. 2026 Jan 29;15(3):1077. doi: 10.3390/jcm15031077 (PMC12898145; doi:10.3390/jcm15031077)
Supplement: Supplementary file 1 [file jcm-15-01077-s001.zip › jcm-4106553-supplementary.pdf]

**Table S1. Sensitivity Analysis Restricted to First IVF Cycle per Woman**<br><br>**N ≈ 1561 women**			
**Variable**	**OR**	**95% CI**	**p**
Female age (per year)	0.960	0.939 – 0.981	<0.001
≥1 high-quality embryo transferred	2.69	1.75 – 4.13	<0.001
Day 5 vs Day 3 transfer	1.41	1.12 – 1.77	0.003
Endometrial thickness (per mm)	1.07	1.02 – 1.12	0.002

This sensitivity analysis was performed to address potential within-patient correlation due to repeated cycles.

Model discrimination:

- **AUC = 0.635 95% CI: 0.614-0.675**

**Table S2. Clustered Marginal Model Using Generalized Estimating Equations (GEE)**			
**Variable**	**OR**	**Robust 95% CI**	**p**
Female age (per year)	0.958	0.939 – 0.977	<0.001
≥1 high-quality embryo transferred	2.71	1.82 – 4.05	<0.001
Day 5 vs Day 3 transfer	1.39	1.14 – 1.71	0.001
Endometrial thickness (per mm)	1.08	1.04 – 1.13	<0.001
*Robust standard errors were used to account for within-patient correlation.			

Table S3. Model Discrimination by Embryo Transfer Type		
**Transfer type**	**N**	**AUC (95% CI)**
Fresh embryo transfer	~380	0.63 (0.61-0.68)
Frozen embryo transfer	~1640	0.64 (0.61-0.67)

Table S4. Bootstrap internal validation results for model discrimination	
	**Value**
Apparent AUC	0.64
95% CI (bootstrap percentile)	0.61 – 0.66
Mean optimism	0.006
Optimism-corrected AUC	0.63
Bootstrap resamples	500
